# The effect of motivational interview based on WhatsApp on the psychological domains of quality of life in infertile women with pcos: A randomized clinical trial

**DOI:** 10.1192/j.eurpsy.2021.2086

**Published:** 2021-08-13

**Authors:** F. Ansari, Z. Hamzehgardeshi, F. Elyasi, M. Moosazadeh, I. Ahmadi

**Affiliations:** 1 Nursing And Midwifery Faculty, Mazandaran University of Medical Sciences, sari, Iran; 2 Neurology, School Of Medicine Psychiatry And Behavioral Sciences Research Center Addiction Research Institutes Sari Imam Khomeini Hospital, Mazandaran University of Medical Sciences, sari, Iran; 3 Health Sciences Research Center Addiction Research Institutes, Mazandaran University of Medical Sciences, sari, Iran; 4 Department Of Obstetrics And Gynecology., School Of Medicine Sexual And Reproductive Health Research Center Sari Imam Khomeini Hospita, Mazandaran University of Medical Sciences, SARI, Iran

**Keywords:** Infertile women, polycystic ovary syndrome, motivational interview, quality of life, psychosis

## Abstract

**Introduction:**

Polycystic ovary syndrome(Pcos) disease significantly decreased quality of life for women. Mental health is one of the components affecting the quality of life of these patients that attention to it is necessary to improve their quality of life.

**Objectives:**

The present study was conducted to determine The effect of Motivational Interview Based on WhatsApp on the Psychological Domains of Quality of Life in Infertile Women with PCOS.

**Methods:**

This randomized controlled clinical trial enrolled 60 Infertile Women with PCOS from the city of Sari-Iran in 2020. Participants were assigned to MI and control groups using block randomization. The intervention group received 5 weekly of MI online via WhatsApp. While the control group received only routine care. The psychological Domains score of quality of life in these individuals was measured using the quality of life questionnaire of polycystic ovary syndrome(MPCOSQ) before and after the intervention. Then, the data were entered into the SPSS software, version 25 and were analyzed using descriptive statistics, chi-square test, t-test, and repeated measures analysis of variance.

**Results:**

No significant difference was observed between the two groups before the intervention mean The Psychological Domains scores (p>0.05). After the intervention, mean (SD) of The Psychological Domains score was 34.8 (11.8) in the intervention group and 30.7 (11.6) in the control.No significant Increasing in the mean between the two groups. The effect size(0.35) was calculated.

**Conclusions:**

The results of the study showed that motivational interviewing is effective in improving the quality of life of women with pcos.

**Disclosure:**

No significant relationships.

